# I love you no matter what: Negative relationship quality and daily encounters in the parent–child tie and their implications for daily mood across the lifespan

**DOI:** 10.1111/famp.13094

**Published:** 2025-01-08

**Authors:** Jane M. Stephenson, Angela Turkelson, Karen L. Fingerman, Kira S. Birditt

**Affiliations:** ^1^ Institute for Social Research University of Michigan Ann Arbor Michigan USA; ^2^ Department of Human Development and Family Sciences University of Texas at Austin Austin Texas USA

**Keywords:** daily experiences, mood, parent–child tie, relationship quality

## Abstract

Parent–child relationship quality has critical implications for parental emotional well‐being across the lifespan. The present study assessed how relationship quality is related to daily encounters between parents and children, how those encounters are linked with parents' mood, and how these associations vary by age. Participants (*N* = 129, ages 33–91) reported baseline relationship quality with a total of 337 children (ages 1–69). In ecological momentary assessments, participants reported encounters with their children and their mood every 3 h for 4 days (*N* = 2220). Analyses revealed that relationship quality was not associated with whether parents had contact with their children. More negative relationship quality was positively associated with unpleasant encounters and negatively associated with pleasant encounters with children. Pleasant encounters with a child were associated with a more positive mood regardless of relationship quality. Unpleasant encounters were associated with a more negative mood, particularly when parents had a more negative relationship with their children. These associations varied significantly by age. Parents were more likely to have contact with more irritating adolescent children than less irritating adolescents and were less likely to have unpleasant encounters with children in emerging adulthood compared to childhood. Older parents' moods were not as strongly associated with unpleasant encounters, though the likelihood of experiencing unpleasant encounters was more closely tied to relationship quality for older parents than younger parents. These findings have important implications for understanding the relationship between family conflict and emotional well‐being across the life course.

Because the parent–child tie is one of the longest lasting and most emotionally intense social relationships, the quality of this tie has important implications for parents' emotional well‐being. Existing literature suggests that close relationships, such as the parent–child tie, are among the most irritating, yet may still involve frequent contact at all ages (Fingerman et al., 2004). Contact between parents and children is characterized by pleasant and unpleasant daily encounters that influence positive and negative moods (Fingerman et al., 2016; Larson & Richards, 1994; Repetti et al., 2011). Literature shows that negative aspects of relationships have a stronger association with health and well‐being than positive aspects (Brooks, 2015; Seeman et al., 2014; Sneed & Cohen, 2014). There is mixed evidence on how negative relationship quality changes across the lifespan. Some theories suggest that relationships may become less negative over time as individuals become more invested in maintaining emotionally meaningful and close ties (Carstensen, 2021), while other studies have found that some relationships remain negative or even become more irritating as people age (Birditt et al., 2020). Parents who have more negative relationships with their children may be particularly at risk for mental and physical health problems as they grow older because negative relationships and emotions tend to be more closely related to deleterious emotional and physical health outcomes (Newsom et al., 2003, 2008; Rook et al., 2004).

This study examined how negative relationship quality in the parent–child tie was associated with contact, valence of encounters in daily life, and parents' moods, as well as how these associations vary among parents and children across different age groups.

## Theoretical background

There are a multitude of theories regarding how relationship quality, daily encounters, and mood are related within the parent–child tie. The intergenerational solidarity model is a mechanistic model of how different aspects of the relationship (e.g., contact, support, and affection) between parents and children are associated (Bengtson & Roberts, 1991; Fingerman et al., 2013). The intergenerational solidarity model posits that affection (i.e., positive feelings) within the parent–child tie is associated with contact between members of the tie. However, more research has found that contact is not always related to positive relationship quality (Guo et al., 2012; Silverstein & Bengtson, 1997; Van Gaalen & Dykstra, 2006). Rather than contact alone, relationship quality in the parent–child tie may be associated with the nature and role of being a parent. Examining how affectional solidarity varies by generation, scholars argue that parents have a greater “intergenerational stake” in their children's lives, which causes them to view their relationships in a more positive light than their children (Birditt & Fingerman, 2013; Birditt, Miller, et al., 2009; Giarrusso et al., 2005). Thus, parents may perceive their relationships as generally less negative even if they are characterized by low contact or unpleasant encounters with their children.

Alternatively, the conflict perspective proposes that parents will have contact equally with children with whom they have more and less negative relationships because irritations are a normative aspect of parent–child relationships across the lifespan, and indeed, may even arise from contact (Birditt & Fingerman, 2013). The conflict perspective posits that disagreements or arguments often arise via daily encounters. Thus, parents may feel more irritated with children with whom they have more daily encounters and report poorer relationship quality with such children (Birditt, Rott, et al., 2009; Fingerman, 2001). Alternatively, parents may reach out more often to support children with problems (Byers et al., 2008; Fingerman et al., 2009; Pillemer & Suitor, 1991a, 1991b) and typically report more negative quality relationships when trying to provide support to children with problems (Birditt et al., 2010). Indeed, research on adolescence in particular finds that parent–child relationships with more conflict tend to be related to more optimal relationship quality (Adams & Laursen, 2007; Branje, 2018). Based on the varying and contrasting theories and research, it seems that parents have contact with their children regardless of their relationship quality. The valence of these encounters may vary by relationship quality, with pleasant encounters with children occurring irrespective of relationship quality, but unpleasant encounters being more likely to occur with children with whom parents have a more negative relationship than those with whom they have a less negative relationship.

Attachment theory posits that social competency and interpersonal trust first form between a child and their primary caregiver and can impact how they build relationships and navigate social situations throughout their lives (Bowlby, 2005). Thus, the attachment style formed between a parent and their child may inform their relationship quality, daily contact, and the valence of their encounters at all ages. Across childhood, positive interactions between the parent and child are important for establishing a healthy attachment (Posada & Lu, 2011). Two studies of attachment and relationship quality in adolescents and their parents found that attachment‐related anxiety but not avoidance predicted parent–child relationship quality (Chow et al., 2017; Walsh & Zadurian, 2023). Thus, anxiously attached parent–child relationships, which tend to involve more unpleasant encounters, may be more closely related to negative relationship quality. Conversely, parent–child relationships with an avoidant attachment characterized by low contact may not have a significant association with the quality of that relationship. A study of parents and their adult children also found that dimensions of attachment had a nuanced association with parental well‐being: high‐quality relationships with children were associated with improved parental well‐being, while increased involvement within the tie in the form of instrumental support provided to parents was associated with poorer well‐being outcomes (Merz et al., 2009).

Other theories explore how factors, besides relationship quality and contact, are involved in the parent–child tie. The family systems perspective on family relationships accounts for how personal characteristics shape the nature of family ties (Norris et al., 2002; Vangelisti, 2021). Thus, the association between relationship quality and contact within the parent–child tie may vary by age, gender, or race. Socioemotional selectivity theory posits that as people age, their future time horizons shorten (Carstensen, 2021). As a result, they experience an increased desire to maintain their close relationships and end up focusing more on the positive aspects of their relationships and less on the negative aspects. Following this line of reasoning, parent–child relationships in later life may experience a “positivity effect” and parents may report fewer unpleasant interactions, perceive their relationships as less negative, and report more positive and less negative moods as they and their children grow older.

## Literature review

### Negative relationship quality, contact, and daily mood in the parent–child tie

Qualities of parent–child relationships may be associated with the types of daily experiences parents have. Parents can have strong connections and frequent contact with children with whom they have negative relationships. Studies testing the conflict perspective and investment models have found that parents did indeed have contact with their children regardless of how negatively they perceived their relationships (Birditt et al., 2020; Fingerman et al., 2004) and reported both pleasant and unpleasant encounters within that contact that were independent of negative relationship quality (Branje, 2018; Fingerman, 2000). Birditt et al. (2010) found that parents experienced similar levels of affection for children with problems as they did for children who were successful but experienced more conflict with their children suffering problems. Along these lines, the frequency of contact with children may not vary by negative relationship quality. Similarly, parents may have equally pleasant daily encounters with children with whom they have both more and less negative relationship quality but may report more unpleasant encounters with children with whom they have a more negative relationship.

Studies show that daily encounters with children of all ages have associations with parents' moods (Fingerman et al., 2016; Larson & Richards, 1994; Repetti et al., 2011). Parents tend to feel more positive when they are with their children than when they are not (Negraia & Augustine, 2020) and positive daily experiences with children tend to be positively related to parent's positive mood (Shoshani & Yaari, 2022). However, negative relationships and unpleasant encounters tend to be more closely associated with mood than less negative relationships and pleasant encounters because the negative aspects are usually more intense and infrequent (Newsom et al., 2003; Rook et al., 2004). Indeed, Fingerman et al. (2016) found that parents reported greater negative emotional well‐being on days when they had unpleasant encounters with their children, but negative encounters were less frequent than positive ones. While research has explored how relationship quality is associated with daily encounters and then how those encounters are associated with parental well‐being, there has been less consideration of how overall perceptions of relationship quality function in conjunction with the valence of daily encounters to explain changes in daily mood among parents at varying life stages.

### Age differences

Overall perceptions of negative relationship quality as well as the frequency and valence of encounters and their implications for well‐being may vary by the life stage of the parent and child. Past research has supported the family systems' perspective and socioemotional selectivity theory and shown that there are age differences in negative relationship quality, contact, daily encounter valence, and mood within the parent–child tie (Birditt & Fingerman, 2013; Noack & Buhl, 2004; Norris et al., 2002).

Parents' negative feelings about their relationships with their children may vary across the life course of those children. A study of parental relationships with children ranging from 0 to 22 years of age found that parents experienced the most satisfaction from their relationships with children when they were under 5 years of age compared to school‐age or adolescent children (Nomaguchi, 2012). Most research on parent–child relationships with younger children considers how parental factors such as adaptation to parenthood demands and parenting style contribute to the quality of the relationship (Noack & Buhl, 2004). As children move into early adulthood and support exchanges become more bidirectional, relationship quality may improve (Noack & Buhl, 2004). As children move into middle adulthood and potentially step into more caring roles for their parents, some studies have found that relationship quality remains generally positive (Fingerman, 2000), while others have found that the parent–child tie experiences more tensions as children juggle competing responsibilities and discrepant expectations within the relationship (Birditt, Miller, et al., 2009).

Contact between parents and children also varies across the life stages. Because parents usually live with and are the primary caregivers for their minor children, there is likely to be little variation by age in the amount of contact between parents and their children under 18, though studies find that as children enter adolescence and early adulthood, they seek more independence from their parents (Noack & Buhl, 2004) and may have less contact with them. Studies also show that parents tend to be more involved in the lives of younger adult children than older adult children (Fingerman et al., 2016), but still have frequent contact with their adult children at all ages (Birditt et al., 2017; Fingerman et al., 2012; Swartz, 2009).

In terms of how the valence of daily encounters in conjunction with perceived negative relationship quality may change with age, Nomaguchi (2012) also argues that while daily interpersonal strains occur between parents and children across the lifespan, the nature of these strains and their associations with overall relationship quality and mood vary by the child's age. Negative experiences with younger children contribute less to relationship quality and daily well‐being than school‐age and adolescent children because those strains have less to do with the children themselves and more with the parenting role. However, while research has found that conflict was more common between parents and adolescent children as compared to younger children, it was not necessarily associated with poorer overall relationship quality (Branje, 2018). Negative encounters between adult children and parents are relatively infrequent and are not necessarily determined by negative relationship quality (Birditt et al., 2010, 2017).

Other research has examined how parental age is associated with relationships and encounters within the parent–child tie. Older individuals tend to have fewer unpleasant daily encounters and have less negative overall relationships than younger adults (Akiyama et al., 2003; Birditt et al., 2005; Fingerman & Birditt, 2003). Studies have found that older adults also reported fewer unpleasant interactions with their children than younger adults (Akiyama et al., 2003; Birditt et al., 2012; Rossi & Rossi, 1990). Thus, as they get older, the parents of adult children may have less negative relationship quality and fewer unpleasant encounters with their children, as compared to the parents of emerging or young adult children.

In addition to exploring how relationship quality and daily encounters are associated, some studies have explored their association with parents' daily moods. Generally, emotional well‐being improves as people age (Carstensen, 2021) and older adults tend to report fewer unpleasant daily encounters and feel less negative in response to such tensions (Birditt et al., 2005).

### Other factors

Research shows that different demographic factors also play a role in relationship quality, contact, and well‐being and these factors, including gender and race, will be considered as covariates in the models for this study. Mothers tend to have more frequent contact and greater positive and negative relationship quality with grown children than fathers across generations (Chai et al., 2020; Fingerman et al., 2020; Rossi & Rossi, 1990). Parents tend to be closer to and interact more with their daughters than their sons (Fingerman et al., 2020; Suitor et al., 2006), although more recent research suggests that daughters' and sons' roles may be converging (Fingerman & Birditt, 2011). African American families report stronger intergenerational ties than European American families and stronger reactions to daily experiences with family members (Chatters et al., 2002; Cichy et al., 2012, 2013).

## The present study

Literature regarding the parent–child tie often examines relationships with either young or adult children rather than examining how daily parent–child interactions and their associations with parents' well‐being vary across age groups (Birditt et al., 2017; Noack & Buhl, 2004). Further, we know little about how negative perceptions of the tie are associated with daily encounters and whether those encounters in conjunction with negative perceptions of relationship quality are associated with well‐being. In this study, we examined variation in negative parent–child relationship quality and how that was associated with the frequency and valence of encounters in daily life, focusing on how these associations vary across children ranging in age from childhood to adulthood. We also examined age differences in the links between negative relationship quality, daily parent–child encounters, and parents' well‐being. We address the following questions:

1. Does negative relationship quality predict contact between parents and children?
2. Does negative relationship quality predict the valence of daily encounters between parents and children?
3. Is the valence of daily encounters and negative relationship quality between parents and children associated with a parent's daily mood?
4. How are these links moderated by parent and child age?

## METHOD

### Participants

Participants were from the Stress and Wellbeing in Everyday Life (SWEL) Study, which was drawn from the longitudinal Social Relations Study (SRS; T. Antonucci, PI) that surveyed individuals in the Detroit tri‐county area. Data for the SWEL Study were collected from March 2018 to March 2020. All study procedures were approved by the University of Michigan Institutional Review Board. A total of 238 adults completed baseline interviews. Of the 238 participants who completed the baseline, 190 reported having any living children and 182 (76%) reported at least one child in their social convoy. This study focused on those participants who reported children in their social networks during their baseline interview and completed at least one ecological momentary assessment (EMA) survey. Of the 182 participants who listed a child in their social network, 129 completed at least one EMA. Among these participants, 50% were Black, 32% were male, and 63% were married or living with a partner. Participants had an average age of 53.85 (SD = 12.29, range = 33–91) and an average of 14 years of schooling, ranging from 10 to 17+ years (Table 1).

**TABLE 1:** Sample characteristics.

	%	*n*
Black (Parent)	50.39	65
Male (Parent)	31.78	41
Male (Child)	49.29	166
Married	62.79	81
Child life stage		
Childhood	17.51	59
Adolescence	11.28	38
Emerging adulthood	19.58	66
Young adulthood	37.39	126
Adulthood	13.06	44
Encounter valence		
Pleasant	92.74	1086
Unpleasant	13.75	161
More negative relationship quality	44.81	151

	*M* (SD)	Range
Age (Parent)	53.85 (12.29)	33–91
Age (Child)	25.61 (12.96)	1–69
Number of children	2.86 (1.92)	1–16
Years of education	14.27 (1.90)	10–17+
Mood		
Positive	3.19 (0.77)	1.13–4.88
Negative	1.32 (0.41)	1–3.86

### Procedure

#### Baseline assessment

Participants completed baseline interviews either in their homes or over the phone in which they reported demographic covariates along with questions about their relationships with their children. To organize their relationships, respondents were asked to place close individuals into three concentric circles representing different levels of closeness (Antonucci & Akiyama, 1987). They were able to list 20 people per circle, for a total of 60 network members. Network sizes for the sample in this study ranged from 3 to 39. The people they could not imagine life without were placed in the first circle followed by less close individuals who were still important in the second and third circles. Participants were then asked specific questions about their network members. They reported the ages of the first 20 members they listed and their perceived relationship quality with the first 10.

#### Ecological momentary assessment

Participants completed surveys sent to them five times per day, every 3 h, from 9 a.m. to 9 p.m. for up to 5 days on mobile study phones. Most participants had 4 days of data, though some were extended to a fifth day if any issues arose. All available data were used in the analyses for this project. In these surveys, participants were asked about their social encounters in the last 3 h and their mood at the time of each survey.

### Measures

#### Child and parent age

The ages of the participants' children that they listed in their social networks were grouped into life stages (childhood = 1–12, adolescence = 13–17, emerging adults = 18–25, young adults = 26–39, and adulthood = 40–69). The age ranges for these groupings were selected based on current literature regarding life stages (Arnett, 2000; Balasundaram & Avulakunta, 2023). The age of social network members was only collected for the first 20 network members of each participant's social convoy, but no children were listed outside this range. However, three participants reported a total of four children in their networks for whom age was not collected. Parental age was a continuous variable measured in the number of years since birth at the time of the interview.

#### Covariates

Participants were asked: “Are you White, Black, Native American, Asian, Hispanic, or another race?” If participants indicated multiple race categories, they were asked to indicate the one category that best described their race. The current sample was drawn from individuals who chose the “Black” or “White” categories on these measures from prior waves. For analytic purposes, racial status was coded as 1 (*Black*) and −1 (*White*) in this current study. Gender was coded as 1 (*male*) and −1 (*female*). Participants reported the highest grade of school or year of college they had completed (0–17+ years of school), and their marital status (1 = *married or living with a partner*, −1 = *widowed, divorced, separated, or never married*). At the child level, participants reported whether they lived with each child listed in their networks (1 = *coresident child and* −1 = *non‐coresident child*) and circle location in the social convoy model (1 = *first circle*, 2 = *second circle*, and 3 = *third circle*). To account for how other social encounters within each reporting period could influence parents' mood ratings at the end of each 3‐h period, we also controlled for the number of other social encounters parents had in each 3‐h interval.

#### Negative relationship quality

To measure negative relationship quality of each child listed in their social networks, participants were asked: “How much does [network member] get on your nerves?” from 1, indicating *not at all*, to 5, indicating *a great deal*. Responses were dichotomized with a median split (median = 3), where 1 = *more irritating child* and −1 = *less irritating child*. Relationship quality was only measured for the first 10 network members. There were two participants who each reported one child in their social networks who were not in the first 10 and thus did not have data on relationship quality.

#### Daily contact

Daily contact was assessed by asking participants, “How many people have you interacted with in the last 3 hours? Interactions can include in person, phone, text, email, social media (Facebook, Twitter, Instagram, WhatsApp, Snapchat) or video chat (FaceTime, Skype, Google Hangouts).” Then, participants were presented with a list of all the people they listed in their social networks and were prompted, “With whom did you interact? Check all that apply.” Then, participants were asked, “Of the interactions you had in the last 3 hours, which ones were positive/enjoyable?” “Of the interactions you had in the last 3 hours, which ones were meaningful/sincere/heartfelt?” and “Of the interactions you had in the last 3 hours, which ones were irritating, hurtful, annoying or stressful?” After each question, they were presented with a list of all the names of people with whom they reported a social encounter and could check off each encounter that they felt matched the valence described in the prompt.

For analytic purposes, social interactions with children in the past 3 h were measured with three variables. The first variable indicated whether the participant had any contact with each child listed in their social convoy in each 3‐h period (0 = *no* and 1 = *yes*). The other two variables measured the valence of the contact in terms of whether the interaction was pleasant or unpleasant. A pleasant encounter was considered any encounter the respondent rated “positive or enjoyable” or “meaningful or sincere/heartfelt” (0 = *no pleasant encounter* and 1 = *pleasant encounter*). An unpleasant encounter was considered any encounter the respondent rated “hurtful, annoying or stressful” or encounters in which they “could have felt irritated or annoyed but decided not to” (0 = *no unpleasant encounter* and 1 = *unpleasant encounter*).

#### Mood

Every 3 h during each day of the assessment, participants reported whether they experienced a series of positive and negative emotions in the last 3 h using a version of the PANAS Scale adapted for the present study (Watson et al., 1988). Negative mood was comprised of seven items including worried, tense, irritated, lonely, angry, bored, or sad (*α* = 0.77), rated from 1 (*not at all*) to 5 (*a great deal*). Positive mood included eight emotions: energetic, loved, happy, calm, content, excited, proud, and optimistic (*α* = 0.86), rated from 1 (*not at all*) to 5 (*a great deal*). Their responses were averaged to create scores for positive and negative moods at each 3‐h interval.

### Analytic strategy

We used multilevel models to account for the hierarchical structure of the data. To fit the binary outcomes, contact and encounter valence, we used generalized linear mixed‐effects models. For the continuous outcomes, positive and negative moods, we fit linear mixed‐effects models. These models were run in R (R Core Team, 2023) using the functions glmer() and lmer() from the *lme4* package (Bates et al., 2015). For models using glmer(), we modeled the data with a binomial distribution using the “bobyqa” optimizer (Bates et al., 2022), 100,000 maximum iterations, and an integer scalar of 10. Models using lmer() were fitted via the default restricted maximum likelihood estimation. To predict contact and encounter valence, we used models with two levels: each encounter with each child was nested within participants. We tested three‐level models with each interaction with each child nested in 3‐h periods nested in participants, as well as encounters with children nested within days nested in participants, but there was not significant variance at the 3‐h or day level. Models predicting mood also had two levels: mood at the 3‐h level nested within participants with child characteristics averaged to the level of each parent. A three‐level model including a day level was tested for models predicting mood but was omitted due to lack of variance.

Models predicting the likelihood of having contact with children and the valence of their encounters (pleasant and unpleasant) were tested in two steps, with the second step split into two parts: (1) main effects of negative relationship quality with child; (2a) interactions between negative relationship quality with child and child's life stage; (2b) interactions between negative relationship quality with child and parent age. Covariates included the participant's race, gender, highest education level, and marital status. Child covariates included gender, circle location, and coresident status.

Parent mood was modeled in two steps: (1) main effects of having contact with children and the valence of their encounters and (2) interactions between an encounter with a child and parent age. Because parents reported multiple interactions with children in the same periods that they reported their mood, the parent's age was used as an interaction term to capture life stage differences and aggregate covariates for participants' children (proportion of minor children, proportion of coresident children, and average circle location) were included as covariates in these models along with parent race and gender. To account for how other encounters within each reporting period could be associated with mood, the number of other people with whom contact was reported was also included as a covariate.

Implications for parent mood were further explored by combining unpleasant encounters with children and negative relationship quality with children into a three‐category variable: 0 = no unpleasant encounters with children (*n* = 605), 1 = at least one unpleasant encounter with a more irritating child (*n* = 93), and 2 = at least one unpleasant encounter with a less irritating child (*n* = 23). Because there were very few periods (*n* = 3) in which parents had unpleasant encounters with both more and less irritating children, these were not included as a category. We also combined pleasant encounters with children and negative relationship quality with children into a four‐category variable: 0 = *no pleasant encounters with children* (*n* = 38), 1 = *pleasant encounter with a more irritating child* (*n* = 286), 2 = *pleasant encounter with a less irritating child* (*n* = 304), and 3 = *pleasant encounters with both more and less irritating children* (*n* = 94). These models were also tested in two steps: (1) main effects of encounters with children and (2) interactions between encounters with children and parent age. Covariates included parent race and gender.

Significant interactions with age (either child life stage or parent age) were probed by calculating the simple intercepts and slopes of the predictors and moderators using the methods presented in Bauer, Curran, and Willoughby (Bauer & Curran, 2005; Curran et al., 2006) and the online calculator, two‐way interaction effects in HLM (Preacher et al., 2006). For the simple slopes involving the continuous moderator, parent age, three values were selected: one standard deviation below the mean, the mean, and one standard deviation above the mean. Thus, younger parents are considered those around age 39, middle‐aged parents are those around age 51, and older parents are those around age 62.

## RESULTS

### Descriptives

Of the participants who reported that they had at least one living child (*N* = 190), 42 (22%) omitted one or more children from their close social network and 8 (4%) did not include any of their children. All subsequent descriptives represent the respondents who listed at least one child in their social networks and completed at least one EMA. Participants listed between 1 and 16 children in their networks with an average of 2.86 (SD = 1.92) children. The average age of the 337 children listed as part of the participants' close social networks was 25.61 (SD = 12.96), ranging from 1 to 69 years. Out of the total number of children listed in participant's networks, 279 were in the first circle, 54 were in the second circle, and 4 were in the third circle. Additionally, 137 of the 337 children listed lived with their parents.

Of the 129 who listed children in their social networks and completed at least one EMA, 112 (87%) reported at least one encounter with a child in one of their EMAs. Of the 2220 total EMAs completed by those participants, there were 1171 encounters with a total of 245 (out of 337 possible) children over 724 three‐hour reporting periods. During periods in which parents report at least one encounter with a child, they reported having encounters with around two other people (SD = 1.3, range = 1–8). Parents reported that 93% of these encounters had pleasant aspects, and 14% had unpleasant aspects (Table 1). Participants had an average negative mood of 1.32 (SD = 0.41), indicating little to no feelings of negative mood. Participants reported an average positive mood of 3.19 (SD = 0.77), indicating that they had some feelings of positive mood. Parents reported a total of 23 (3%) 3‐h periods in which they had unpleasant encounters with at least one irritating child, 93 (13%) 3‐h periods in which they had unpleasant encounters with at least one less irritating child, and 605 (84%) periods in which they had no unpleasant encounters with any children. Parents reported 286 (40%) 3‐h periods in which they had at least one pleasant encounter with a more irritating child, 304 (42%) periods in which they had a pleasant encounter with a less irritating child, 94 (13%) periods in which they had pleasant encounters with both more irritating and less irritating children, and 38 (5%) periods in which they did not have any pleasant encounters with any children. In terms of contact by age group, parents reported 421 (36%) encounters with children under 13, 179 (15%) encounters with adolescent children, 174 (15%) encounters with children in emerging adulthood, 273 (23%) encounters with young adult children, and 86 (7%) encounters with children over age 39.

### Negative relationship quality and valence of daily encounters

After controlling for the race, gender, age, education level, and marital status of the parent, as well as each child's gender, circle location, coresident status, and life stage, overall ratings of irritation were not associated with the likelihood of having contact with that child (Table 2).

**TABLE 2:** Encounter valence: Associations between relationship quality and child life stage.

	Contact (*n* = 5657)	Unpleasant encounters (*n* = 1131)	Pleasant encounters (*n* = 1131)
	Odds ratio (Confidence interval)	Odds ratio (Confidence interval)	Odds ratio (Confidence interval)
Step 1			
Parent race	0.93 (0.76, 1.13)	0.98 (0.63, 1.53)	**0.44 (0.22, 0.91)**
Parent gender	0.82 (0.66, 1.02)	0.78 (0.49, 1.25)	0.76 (0.38, 1.69)
Parent age	1.01 (0.99, 1.04)	1.00 (0.93, 1.06)	0.98 (0.86, 1.06)
Education level	1.03 (0.93, 1.15)	1.09 (0.86, 1.41)	0.77 (0.52, 1.24)
Marital status	1.10 (0.88, 1.37)	0.94 (0.58, 1.54)	0.62 (0.23, 1.34)
Circle location	**0.45 (0.34, 0.70)**	1.54 (0.54, 4.37)	0.56 (0.12, 2.67)
Child coresident status	**2.50 (2.16, 2.90)**	0.79 (0.48, 1.29)	1.24 (0.65, 2.34)
Child gender	0.94 (0.86, 1.04)	1.13 (0.88, 1.43)	0.88 (0.57, 1.37)
Child life stage^a^			
Adolescence	**0.45 (0.31, 0.65)**	0.40 (0.14, 1.10)	**0.16 (0.04, 0.87)**
Emerging adulthood	**0.57 (0.36, 0.65)**	**0.22 (0.05, 0.94)**	0.41 (0.04, 3.91)
Young adulthood	**0.52 (0.28, 0.94)**	0.40 (0.06, 2.60)	0.32 (0.02, 5.26)
Adulthood	**0.45 (0.21, 0.96)**	0.13 (0.01, 1.62)	0.84 (0.03, 21.73)
Negative relationship quality	1.04 (0.93, 1.17)	**2.20 (1.58, 3.06)**	**0.53 (0.31, 0.83)**
Step 2a			
Negative relationship quality × adolescence	**1.59 (1.13, 2.23)**	0.59 (0.22, 1.59)	1.22 (0.32, 4.62)
Negative relationship quality × emerging adulthood	0.95 (0.67, 1.34)	1.02 (0.36, 2.87)	1.31 (0.28, 6.15)
Negative relationship quality × young adulthood	1.30 (0.98, 1.72)	1.90 (0.83, 4.38)	0.47 (0.12, 1.74)
Negative relationship quality × adulthood	1.09 (0.70, 1.68)	1.29 (0.31, 5.41)	0.70 (0.12, 4.17)
Step 2b			
Negative relationship quality × parent age	1.00 (0.99, 1.01)	**1.04 (1.00, 1.07)**	0.97 (0.93, 1.01)

*Note*: Steps 2a and 2b included all covariates as in Step 1. Bolded font indicates significance at *p* <0.05. Intervals were calculated with 95% confidence.

^a^ Childhood was the reference group.

Negative relationship quality was significantly associated with the valence of encounters with children. Feeling greater irritation about a child was associated with a lower likelihood of having pleasant encounters and a higher likelihood of having unpleasant encounters with that child in daily life (Table 2).

### Daily contact, encounter valence, and mood

All models measuring daily contact and encounter valence with positive and negative mood as the dependent variables included parent race, gender, age, education level, and marital status as well as aggregate child characteristics including proportion of minor children, proportion of coresident children, and average circle location of all children as covariates. Models also controlled for the number of encounters with other social partners reported within each period. Having an unpleasant encounter with any child regardless of relationship quality was significantly associated with a lower positive mood and a greater negative mood (Table 3). Conversely, having a pleasant encounter with any child regardless of relationship quality was significantly associated with a greater positive mood and a lower negative mood (Table 3).

**TABLE 3:** Daily mood: Associations between contact with child and parent age.

	Contact (*n* = 2215)		Unpleasant encounters (*n* = 716)		Pleasant encounters (*n* = 719)	
	Positive mood	Negative mood	Positive mood	Negative mood	Positive mood	Negative mood
	𝛽 (CI)	𝛽 (CI)	𝛽 (CI)	𝛽 (CI)	𝛽 (CI)	𝛽 (CI)
Step 1						
Parent race	0.04 (−0.08, 0.16)	−0.04 (−0.10, 0.01)	0.08 (−0.05, 0.21)	**−0.05 (−0.1, >−0.01)**	0.09 (−0.04, 0.22)	**−0.06 (−0.11, −0.01)**
Parent gender	−0.02 (−0.15, 0.10)	−0.02 (−0.08, 0.03)	0.04 (−0.10, 0.18)	0.01 (−0.05, 0.06)	0.05 (−0.09, 0.19)	> −0.01 (−0.06, 0.05)
Parent age	0.01 (−0.01, 0.022)	**−0.01 (−0.02, >−0.01)**	<0.01 (−0.02, 0.02)	>−0.01 (−0.01, <0.01)	<0.01 (−0.02, 0.02)	−0.01 (−0.01, <0.01)
Education level	−0.01 (−0.067, 0.054)	<0.01 (−0.02, 0.03)	>−0.01 (−0.07, 0.06)	0.01 (−0.02, 0.03)	>−0.01 (−0.07, 0.06)	0.01 (−0.02, 0.04)
Marital status	−0.02 (−0.15, 0.102)	**−0.10 (−0.16, −0.05)**	−0.03 (−0.17, 0.11)	−0.05 (−0.10, <0.01)	−0.02 (−0.16, 0.12)	**−0.06 (−0.12, >−0.01)**
Prop minor children	0.06 (−0.459, 0.58)	−0.06 (−0.29, 0.17)	−0.27 (−0.85, 0.30)	−0.06 (−0.28, 0.16)	−0.30 (−0.86, 0.27)	−0.05 (−0.29, 0.18)
Prop coresident children	−0.20 (−0.603, 0.199)	**0.19 (0.02, 0.37)**	−0.01 (−0.46, 0.43)	0.10 (−0.07, 0.27)	0.01 (−0.43, 0.45)	0.07 (−0.11, 0.25)
Avg circle location	−0.16 (−0.472, 0.154)	**0.14 (>0.01, 0.28)**	−0.04 (−0.40, 0.31)	−0.09 (−0.22, 0.05)	−0.03 (−0.38, 0.32)	−0.08 (−0.23, 0.06)
Other encounters	**0.04 (0.011, 0.068)**	>−0.01 (−0.02, 0.02)	**0.05 (0.021, 0.09)**	<0.01 (−0.02, 0.03)	**0.06 (0.03, 0.09)**	−0.01 (−0.03, 0.02)
Encounter with child	0.02 (−0.018, 0.052)	>−0.01 (−0.03, 0.02)	**−0.09 (−0.15, −0.04)**	**0.16 (0.13, 0.20)**	**0.22 (0.14, 0.31)**	**−0.09 (−0.16, −0.03)**
Step 2						
Encounter with child × parent age	<0.01 (>−0.01, <0.01)	>−0.01 (>−0.01, <0.01)	<0.01 (−0.01, 0.01)	<0.01 (>−0.01, 0.01)	<0.01 (−0.01, 0.01)	>−0.01 (−0.01, 0.01)

*Note*: Step 2 included all covariates as in Step 1. Bolded font indicates significance at *p* < 0.05. Intervals were calculated with 95% confidence.

The valence of encounters in conjunction with relationship quality had significant associations with parents' moods. Unpleasant encounters with more irritating children were associated with lower positive mood compared to not having any unpleasant encounters. Unpleasant encounters with more irritating children were associated with a greater negative mood than when parents had no encounters with their children. Likewise, having an unpleasant encounter with a less irritating child was also associated with a greater negative mood than having no unpleasant encounters, but was not significantly associated with a positive mood (Table 4).

**TABLE 4:** Daily mood: Associations between relationship quality, daily encounters, and parent age.

	Unpleasant encounters (*n* = 716)		Pleasant encounters (*n* = 717)	
	Positive mood	Negative mood	Positive mood	Negative mood
	𝛽 (CI)	𝛽 (CI)	𝛽 (CI)	𝛽 (CI)
Step 1				
Parent race	0.08 (−0.05, 0.21)	**−0.05 (−0.10, >−0.01)**	0.09 (−0.04, 0.22)	**−0.06 (−0.11, −0.01)**
Parent gender	0.04 (−0.10, 0.18)	0.01 (−0.05, 0.06)	0.05 (−0.09, 0.19)	< 0.01 (−0.05, 0.06)
Parent age	<0.01 (−0.02, 0.02)	<0.01 (−0.01, <0.01)	>−0.01 (−0.02, 0.02)	−0.01 (−0.01, < 0.01)
Education level	>−0.01 (−0.07, 0.06)	0.01 (−0.02, 0.03)	>−0.01 (−0.07, 0.06)	0.01 (−0.02, 0.04)
Marital status	−0.03 (−0.17, 0.11)	−0.05 (−0.10, <0.01)	−0.02 (−0.16, 0.12)	**−0.06 (−0.12, >−0.01)**
Prop minor children	−0.27 (−0.85, 0.30)	−0.05 (−0.27, 0.16)	−0.3 (−0.87, 0.27)	−0.03 (−0.27, 0.20)
Prop coresident children	−0.01 (−0.46, 0.44)	0.1 (−0.07, 0.27)	0.01 (−0.44, 0.45)	0.06 (−0.12, 0.24)
Avg circle location	−0.04 (−0.40, 0.31)	−0.09 (−0.22, 0.05)	−0.02 (−0.37, 0.33)	−0.09 (−0.23, 0.05)
Other encounters	**0.06 (0.02, 0.09)**	<0.01 (−0.02, 0.03)	**0.06 (0.02, 0.09)**	>−0.01 (−0.03, 0.02)
Relationship quality^a^				
More irritating child	**−0.19 (−0.32, −0.07)**	**0.32 (0.23, 0.41)**	**0.45 (0.26, 0.64)**	**−0.15 (−0.29, −0.01)**
Less irritating child	−0.15 (−0.37, 0.06)	**0.35 (0.19, 0.50)**	**0.42 (0.22, 0.62)**	**−0.20 (−0.34, −0.06)**
Both types of children			**0.50 (0.27, 0.73)**	**−0.27 (−0.43, −0.10)**
Step 2				
Relationship quality^a^				
More irritating child × Parent age	>−0.01 (−0.02, 0.01)	**0.01 (<0.01, 0.02**)	<0.01 (−0.02, 0.02)	>−0.01 (−0.02, 0.01)
Less irritating child × parent age	**0.03 (<0.01, 0.05)**	**−0.03 (−0.05, −0.02)**	<0.01 (−0.02, 0.03)	−0.01 (−0.02, 0.01)
Both types of child × parent age			<0.01 (−0.02, 0.02)	<0.01 (−0.01, 0.02)

*Note*: Step 2 included all covariates as in Step 1. Bolded font indicates significance at *p* < 0.05. Intervals were calculated with 95% confidence.

^a^ Reference group was having no contact with any children in a 3‐h period.

Having pleasant encounters with an irritating child, a less irritating child, and both irritating and less irritating children were all significantly associated with greater positive mood and lower negative mood compared to having no encounters with any children (Table 4).

### Differences by child life stage and parent age

**FIGURE 1:**
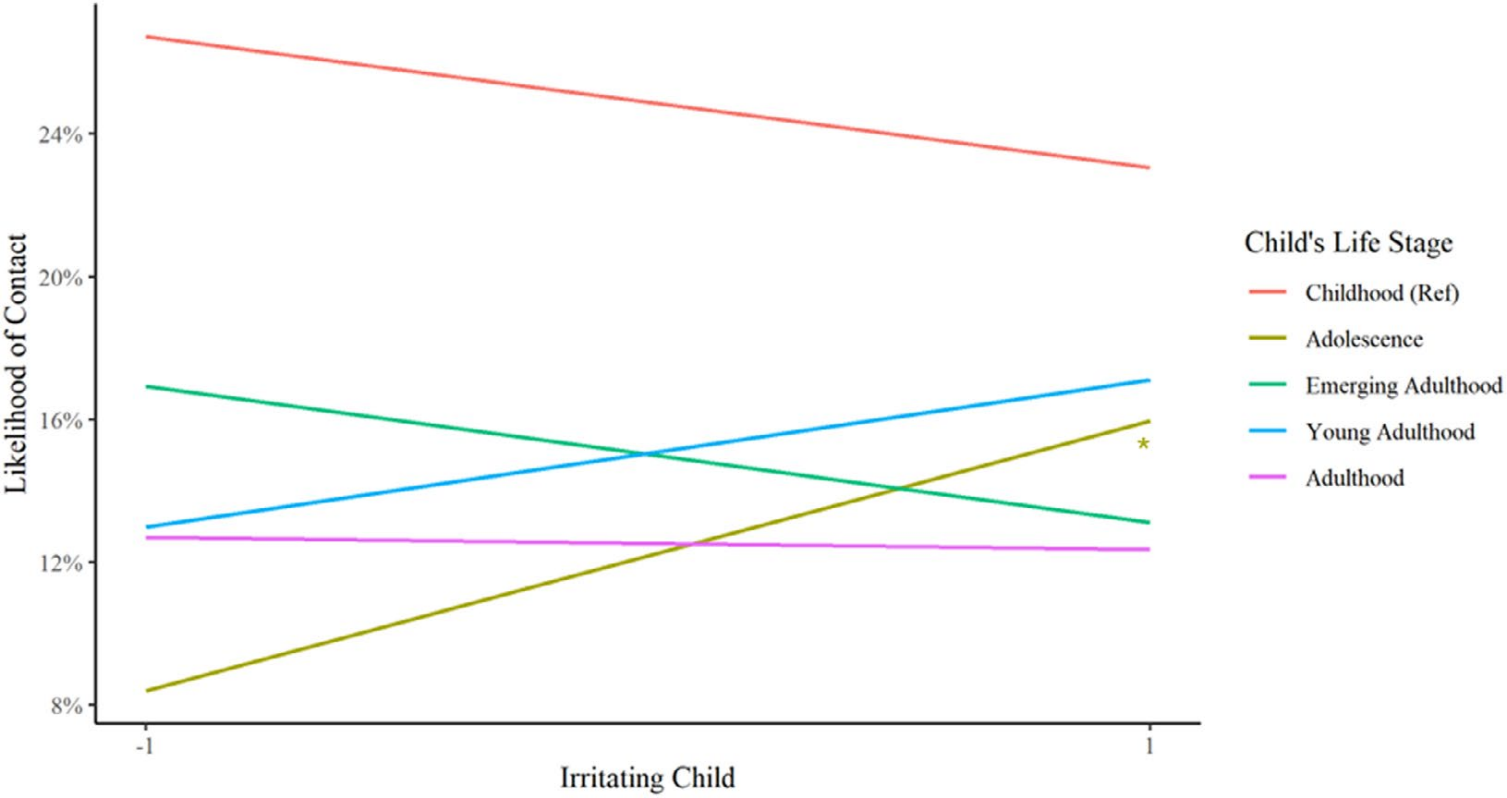
Associations between negative relationship quality and contact with children by child life stage. *p < 0.05.

Models predicting contact revealed both main effects of child age and interactions with negative relationship quality and child age. Parents were significantly less likely to have contact with children in adolescence and beyond as compared to childhood (Table 2). Additionally, the child's life stage significantly moderated the association between negative relationship quality and having contact with a child only if that child was in adolescence (Figure 1). Parents were more likely to have contact with adolescent children that they found irritating than adolescent children that they found less irritating (𝛽 = 0.34, OR = 1.41, and *p* = 0.02).

Models predicting encounter valence revealed main effects for child life stage, but no significant interactions between child life stage and negative relationship quality. Parents were significantly less likely to report an unpleasant encounter (𝛽 = −1.53, OR = 0.22, and *p* = 0.04) with children in emerging adulthood (ages 18–25) compared to younger children (ages 1–12). In terms of pleasant encounters, parents were significantly less likely to have a pleasant encounter (𝛽 = −1.84, OR = 0.16, and *p* = 0.03) with adolescent children (ages 13–17) compared to younger children (ages 1–12).

**FIGURE 2:**
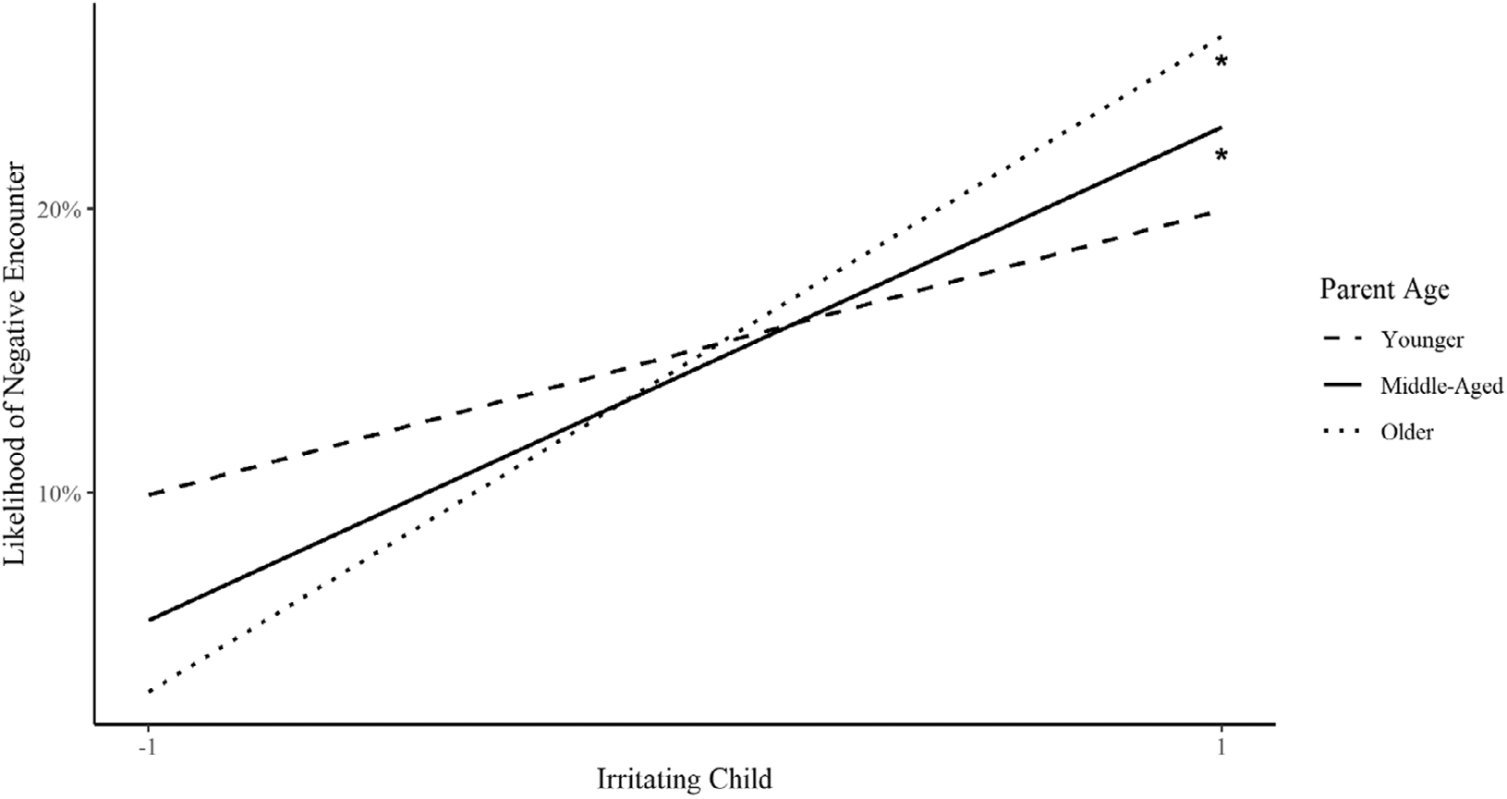
Associations between negative relationship quality and unpleasant encounters with children by parent age. *p < 0.05.

Models also explored parent age as a main effect and moderator of the association between relationship quality and contact. Parent age did not significantly interact with negative relationship quality when predicting contact or pleasant daily encounters. However, parent age did significantly interact with negative relationship quality when predicting unpleasant daily encounters. Middle‐aged and older parents were more likely to have unpleasant encounters with children they found irritating than those they did not find irritating (middle‐aged: 𝛽 = 0.80 OR = 2.23, and *p* < 0.01; older: 𝛽 = 1.21 OR = 3.35, and *p* < 0.01). The association was not significant among younger parents (Figure 2).

**FIGURE 3:**
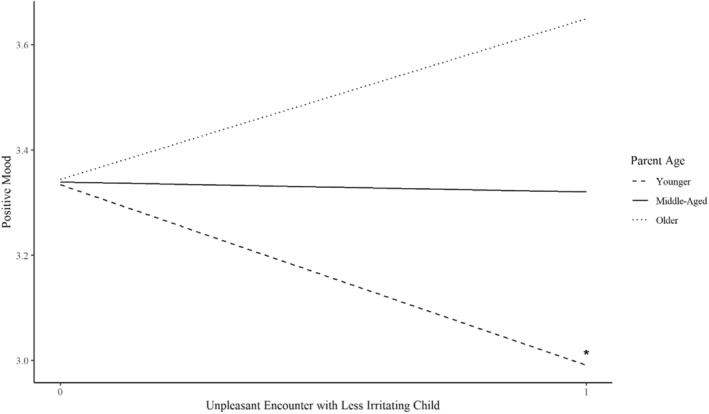
Associations between unpleasant encounters and with less irritating children and parents' positive mood by parent age. *Y*‐axis does not represent full scale (0–5). *p < 0.05.

**FIGURE 4:**
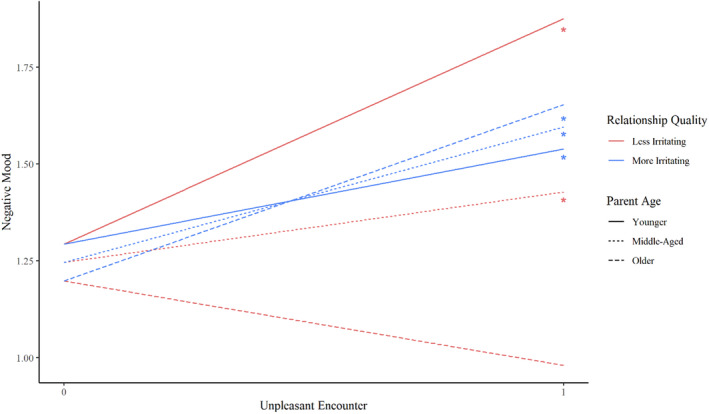
Associations between encounters with less irritating children and parents' negative mood by parent age. *Y*‐axis does not represent full scale (0–5). *p < 0.05.

To predict parent mood, we only were able to examine the effects of parent age rather than child life stage, as parents may have interacted with multiple children across different life stages in each 3‐h period. The age of the parent significantly moderated the association between having an unpleasant encounter with a less irritating child and the parents' positive and negative moods. Having an unpleasant encounter with a less irritating child was significantly associated with lower positive mood among younger parents (𝛽 = −0.34, SD = 0.14, *p* = 0.02), but was not significantly associated with positive mood among middle‐aged or older parents (Figure 3). Having an unpleasant encounter with a less irritating child was significantly associated with a greater negative mood among younger (𝛽 = 0.58, SD = 0.10, *p* < 0.001) and middle‐aged parents (𝛽 = 0.18, SD = 0.09, *p* = 0.04) but not among older parents (Figure 4). Additionally, having an unpleasant encounter with a more irritating child was significantly associated with a greater negative mood across all age groups for parents (younger: 𝛽 = 0.25, SD = 0.06, and *p* < 0.001; middle: 𝛽 = 0.35, SD = 0.05, and *p* < 0.001; older: 0.45, SD = 0.08, and *p* < 0.001).

### Post‐hoc tests

All models considered interactions with parent gender to examine whether the likelihood of interactions and the associations with well‐being varied between mothers and fathers. There was a significant interaction between parent gender and negative relationship quality predicting negative mood. Fathers had significantly lower negative moods after having a pleasant encounter with a child, whereas the association was not significant among mothers. Likewise, fathers had significantly lower negative moods after having pleasant encounters with both more irritating and less irritating children in the same period. There was no significant effect for mothers. The gender of the parent did not significantly moderate the association between having pleasant or unpleasant interactions with either more or less irritating children and parents' positive or negative mood.

Models predicting contact and encounter valence also considered interactions between negative relationship quality and child gender. The gender of the child did not significantly moderate the association between negative relationship quality and contact or daily encounters.

The interaction between parent race and negative relationship quality for predicting contact, encounter valence, and mood was also considered. Parent race did not significantly moderate the association between relationship quality and contact or daily encounter valence. The race of the parent significantly moderated the association between having an unpleasant encounter with any type of child and a negative mood. Black parents had significantly lower negative moods after having an unpleasant encounter with a child compared to White parents (𝛽 = −0.04 and SD = 0.02).

## DISCUSSION

A burgeoning literature shows that negative relationship quality and conflict with children have important implications for parents' well‐being (Brooks, 2015; Newsom et al., 2003; Rook et al., 2004; Seeman et al., 2014; Sneed & Cohen, 2014). However, there is little understanding of the daily mechanisms accounting for these effects. Most literature examines either young children or adult children and does not examine how relationship quality and its implications vary across all life stages. This study examined the parent–child relationship across the age ranges and assessed links among overall negative relationship quality, contact, encounter valence, and parents' mood in daily life.

### Relationship quality and daily contact

Consistent with the literature, this study found that parents had contact with their children regardless of their relationship quality. As finding a child more irritating did not predict less contact with that child, our findings support the conflict perspective of relationships which posits that disagreements or arguments that may contribute to an overall poorer perception of relationship quality are a normative part of the parent–child tie and may even arise from contact (Birditt & Fingerman, 2013). For instance, Van Gaalen and Dykstra (2006) found that solidarity was not predictive of conflict within parent–child relationships. Birditt et al. (2010) also found that higher feelings of ambivalence (both positive and negative perceptions of a relationship) were associated with greater contact.

### Relationship quality and daily encounter valence

While relationship quality had no significant association with the likelihood of having daily encounters between parents and children, the quality of the relationship did predict the valence of daily encounters. Parents were more likely to have unpleasant and less likely to have pleasant encounters with children they perceived as more irritating overall. These findings align with previous research using the conflict perspective that found that parents tend to report more negative quality relationships when trying to support children with problems (Birditt et al., 2010). This contrasts with the findings from Fingerman (2000), who found no association between relationship quality and mothers' descriptions of their most recent enjoyable visits with their daughters. However, a difference in the measurement of relationship quality may account for these differences since the present study only measured negative relationship quality in terms of how irritating the child was to the parent, and the Fingerman study measured relationship quality through dimensions of trust, respect, understanding, fairness, and affection.

### Relationship quality, daily contact, and parent mood

Encounter valence had significant associations with parent mood that were in some cases different depending on negative relationship quality. Irrespective of negative relationship quality, unpleasant encounters with children were associated with a greater negative mood. Unpleasant encounters with more irritating children were associated with lower positive mood. However, unpleasant encounters with less irritating children *did not* predict positive mood, indicating there may be some benefit to having lower negative quality ties. These findings align with previous research that found that unpleasant encounters with more irritating individuals are more closely associated with mood than encounters with those who are less irritating because more negative relationships tend to be more emotionally intense and unpleasant encounters generally occur more infrequently and are thus more emotionally salient (Newsom et al., 2003; Rook et al., 2004).

Pleasant encounters were associated with greater positive and lower negative moods regardless of whether negative relationship quality was considered. These findings indicate that parents receive emotional benefits from positive encounters with their children regardless of their overall negative relationship quality. This is consistent with previous research by Fingerman et al. (2016), who found that parents reported more positive moods along with pleasant daily encounters with their children irrespective of their relationship quality. Overall, the findings from this study regarding contact, encounter valence, and parent's mood align with other research that has also found that parent's mood is associated both positively and negatively with the valence of their daily encounters with their children (Fingerman et al., 2016; Repetti et al., 2011).

### Differences by child life stage and parent age

The associations between negative relationship quality, daily contact, valence of daily encounters, and parent mood varied by the life stage of the child and the age of the parent. Parents were more likely to have contact with adolescent children that they found more irritating than those they did not find irritating. This aligns with previous literature that discusses how conflict with adolescent children is common, but not necessarily detrimental to the parent–child tie (Adams & Laursen, 2007; Branje, 2018). This could also be because parents are more responsible for their children when they are minors and must intervene more when an adolescent is having problems, whereas they do not have to be as involved with children whom they find irritating when those children are adults.

The present study also found the valence of daily encounters varied by parent age. Middle‐aged and older parents but not younger parents were more likely to report unpleasant encounters with irritating children than with less irritating children. This could be because older parents may reach out to their adult children to discuss problems in the relationship that may lead to unpleasant encounters, while younger parents of minor children are more heavily involved in the lives of their children regardless of relationship quality and thus their unpleasant encounters with their children are less associated with their relationship quality. Past research shows that relationship quality is less dependent on encounters with young children because children have less agency in determining the valence of the encounter than an adult child (Noack & Buhl, 2004).

The associations between daily interactions with children and parents' moods varied by parents' age. Unpleasant encounters with less irritating children were associated with lower positive moods among younger parents but not among middle‐aged and older parents. Likewise, negative mood was positively associated with having an unpleasant encounter with an irritating child for younger and middle‐aged parents but not older parents. This aligns with previous literature that suggests that in general, emotional well‐being improves with age as individuals become more aware of their limited time and shift their priorities to more meaningful pursuits (Carstensen, 2021). Additionally, the present findings align with research on changes in parental well‐being across different ages. Studies have found that older parents report less emotional distress than younger parents because younger parents are likely to be caring for minor children, which comes with high emotional demands (Nomaguchi, 2012; Schiffman et al., 2014). Conversely, older parents can invest less emotional energy when their children are older and more independent (Evenson & Simon, 2005).

As older and middle‐aged parents experience more unpleasant encounters with irritating children but experience less negative change in their mood than younger parents, the findings of this study suggest that different age groups of parents experience the highs and lows of parenting differently and may require different forms of support.

### Limitations and future directions

As with any study, this one had its limitations. First, there were methodological limitations in the measurement of relationship quality. This study measured relationship quality in terms of how much each child got on their nerves. In future studies, expanded measures could better capture the nuances of relationships and encounters. Secondly, the ecological momentary assessment component of this project only lasted for a maximum of 5 days with most participants completing 4 days, which may not be long enough to accurately capture an individual's typical life. For instance, the present study may not have captured infrequent contact between parents and children who live far apart. An extended daily assessment or follow‐up study would better capture typical encounters within the parent–child relationship. Because the sample was taken only from individuals from an existing longitudinal study that began in the Detroit area, the findings may have limited generalizability to the greater population. Future studies with a larger national sample may be more representative of the population.

Additionally, this study did not consider how emotion regulation strategies, coping mechanisms, and conflict responses moderate the associations between daily encounters and mood. There are, of course, many ways to deal with unpleasant interactions. Future research could explore how coping moderates the link between unpleasant encounters with children and parental emotional well‐being. The analyses in this study were also limited to parent's relationships with children they listed in their social networks. Fifty participants omitted at least one child from their networks. Thus, we were not able to explore how relationship quality with these children, which is likely to be poor due to their omission from parents' social networks, was related to daily contact and well‐being. Future research could prompt parents to report relationship quality with all of their children regardless of whether they consider them in their close networks. Lastly, while the present study accounted for the presence of other social encounters within the same reporting period, it could not definitively tease out whether mood during each 3‐h period was directly related to encounters with children. To remedy this, a future project could prompt participants to record their mood after each social encounter. Incorporating biological measures in future studies could elucidate how physiological reactivity is related to unpleasant daily encounters with children. Longitudinal studies could also explore the long‐term associations of negative relationship quality and unpleasant daily encounters with emotional and physical well‐being.

Overall, this study indicates that negative relationship quality in the parent–child tie has important implications for daily interactions and mood across the lifespan. Practitioners can use these findings to help parents better understand how conflict is a normative part of the parent–child tie at all ages, be aware of the emotional implications of their encounters with their children, and contextualize how those implications will change across the lifespan of both them and their children.

## Data Availability

Data and additional documentation of sample and measures are available upon request to the corresponding author.
